# Transdiagnostic dimensions of psychopathology at first episode psychosis: findings from the multinational EU-GEI study

**DOI:** 10.1017/S0033291718002131

**Published:** 2018-10-04

**Authors:** Diego Quattrone, Marta Di Forti, Charlotte Gayer-Anderson, Laura Ferraro, Hannah E Jongsma, Giada Tripoli, Caterina La Cascia, Daniele La Barbera, Ilaria Tarricone, Domenico Berardi, Andrei Szöke, Celso Arango, Antonio Lasalvia, Andrea Tortelli, Pierre-Michel Llorca, Lieuwe de Haan, Eva Velthorst, Julio Bobes, Miguel Bernardo, Julio Sanjuán, Jose Luis Santos, Manuel Arrojo, Cristina Marta Del-Ben, Paulo Rossi Menezes, Jean-Paul Selten, EU-GEI WP2 Group, Peter B Jones, James B Kirkbride, Alexander L Richards, Michael C O'Donovan, Pak C Sham, Evangelos Vassos, Bart PF Rutten, Jim van Os, Craig Morgan, Cathryn M Lewis, Robin M Murray, Ulrich Reininghaus

**Affiliations:** 1 Social, Genetic and Developmental Psychiatry Centre, Institute of Psychiatry, Psychology and Neuroscience, King's College London, London SE5 8AF, UK; 2 National Institute for Health Research (NIHR) Mental Health Biomedical Research Centre at South London and Maudsley NHS Foundation Trust and King's College London, UK; 3 Department of Health Service and Population Research, Institute of Psychiatry, King's College London, De Crespigny Park, Denmark Hill, London SE5 8AF, UK; 4 Department of Experimental Biomedicine and Clinical Neuroscience, University of Palermo, Via G. La Loggia 1, 90129 Palermo, Italy; 5 Department of Psychiatry, University of Cambridge, Herchel Smith Building for Brain & Mind Sciences, Forvie Site, Robinson Way, Cambridge, CB2 0SZ, UK; 6 Department of Psychosis Studies, Institute of Psychiatry, King's College London, De Crespigny Park, Denmark Hill, London SE5 8AF, UK; 7 Department of Medical and Surgical Science, Psychiatry Unit, Alma Mater Studiorum Università di Bologna, Viale Pepoli 5, 40126 Bologna, Italy; 8 INSERM, U955, Equipe 15, 51 Avenue de Maréchal de Lattre de Tassigny, 94010 Créteil, France; 9 Department of Child and Adolescent Psychiatry, Hospital General Universitario Gregorio Marañón, School of Medicine, Universidad Complutense, IiSGM (CIBERSAM), C/Doctor Esquerdo 46, 28007 Madrid, Spain; 10 Section of Psychiatry, Azienda Ospedaliera Universitaria Integrata di Verona, Piazzale L.A. Scuro 10, 37134 Verona, Italy; 11 Etablissement Public de Santé Maison Blanche, Paris 75020, France; 12 Université Clermont Auvergne, EA 7280, Clermont-Ferrand 63000, France; 13 Department of Psychiatry, Early Psychosis Section, Academic Medical Centre, University of Amsterdam, Meibergdreef 5, 1105 AZ Amsterdam, The Netherlands; 14 Department of Medicine, Psychiatry Area, School of Medicine, Universidad de Oviedo, Centro de Investigación Biomédica en Red de Salud Mental (CIBERSAM), C/Julián Clavería s/n, 33006 Oviedo, Spain; 15 Barcelona Clinic Schizophrenia Unit, Neuroscience Institute, Hospital clinic, Department of Medicine, University of Barcelona, IDIBAPS, CIBERSAM, Barcelona, Spain; 16 Department of Psychiatry, School of Medicine, Universidad de Valencia, Centro de Investigación Biomédica en Red de Salud Mental (CIBERSAM), C/Avda. Blasco Ibáñez 15, 46010 Valencia, Spain; 17 Department of Psychiatry, Servicio de Psiquiatría Hospital “Virgen de la Luz”, C/Hermandad de Donantes de Sangre, 16002 Cuenca, Spain; 18 Department of Psychiatry, Psychiatric Genetic Group, Instituto de Investigación Sanitaria de Santiago de Compostela, Complejo Hospitalario Universitario de Santiago de Compostela, Spain; 19 Division of Psychiatry, Department of Neuroscience and Behaviour, Ribeirão Preto Medical School, University of São Paulo, São Paulo, Brazil; 20 Department of Preventative Medicine, Faculdade de Medicina FMUSP, University of São Paulo, São Paulo, Brazil; 21 Rivierduinen Institute for Mental Health Care, Sandifortdreef 19, 2333 ZZ Leiden, The Netherlands; 22 Department of Psychiatry and Neuropsychology, School for Mental Health and Neuroscience, South Limburg Mental Health Research and Teaching Network, Maastricht University Medical Centre, P.O. Box 616, 6200 MD Maastricht, The Netherlands; 23 CAMEO Early Intervention Service, Cambridgeshire & Peterborough NHS Foundation Trust, Cambridge, CB21 5EF, UK; 24 Psylife Group, Division of Psychiatry, University College London, 6th Floor, Maple House, 149 Tottenham Court Road, London W1T 7NF, UK; 25 Division of Psychological Medicine and Clinical Neurosciences, MRC Centre for Neuropsychiatric Genetics and Genomics, Cardiff University, Cardiff CF24 4HQ, UK; 26 Department of Psychiatry, the University of Hong Kong, Hong Kong, China; 27 Centre for Genomic Sciences, Li KaShing Faculty of Medicine, The University of Hong Kong, Hong Kong, China; 28 Brain Centre Rudolf Magnus, Utrecht University Medical Centre, Utrecht, The Netherlands; 29 Central Institute of Mental Health, Medical Faculty Mannheim, University of Heidelberg, Mannheim, Germany

**Keywords:** Bifactor model, diagnostic categories, first episode psychosis, psychopathology, symptom dimensions

## Abstract

**Background:**

The value of the nosological distinction between non-affective and affective psychosis has frequently been challenged. We aimed to investigate the transdiagnostic dimensional structure and associated characteristics of psychopathology at First Episode Psychosis (FEP). Regardless of diagnostic categories, we expected that positive symptoms occurred more frequently in ethnic minority groups and in more densely populated environments, and that negative symptoms were associated with indices of neurodevelopmental impairment.

**Method:**

This study included 2182 FEP individuals recruited across six countries, as part of the EUropean network of national schizophrenia networks studying Gene–Environment Interactions (EU-GEI) study. Symptom ratings were analysed using multidimensional item response modelling in M*plus* to estimate five theory-based models of psychosis. We used multiple regression models to examine demographic and context factors associated with symptom dimensions.

**Results:**

A bifactor model, composed of one general factor and five specific dimensions of positive, negative, disorganization, manic and depressive symptoms, best-represented associations among ratings of psychotic symptoms. Positive symptoms were more common in ethnic minority groups. Urbanicity was associated with a higher score on the general factor. Men presented with more negative and less depressive symptoms than women. Early age-at-first-contact with psychiatric services was associated with higher scores on negative, disorganized, and manic symptom dimensions.

**Conclusions:**

Our results suggest that the bifactor model of psychopathology holds across diagnostic categories of non-affective and affective psychosis at FEP, and demographic and context determinants map onto general and specific symptom dimensions. These findings have implications for tailoring symptom-specific treatments and inform research into the mood-psychosis spectrum.

## Introduction

Current nosology classifies the observed manifestations of psychosis into two main categories of non-affective (e.g. schizophrenia, schizoaffective disorder) and affective psychosis (e.g. bipolar and major depressive disorders with psychotic features) (World Health Organization, 1992; American Psychiatric Association, 2013). However, the scientific accessibility of discrete ‘natural disease entities’ in psychiatry has been questioned since Kraepelin's original distinction between dementia praecox and manic-depressive psychosis (Kraepelin, 1899; Murray *et al*., 2004; Craddock and Owen, 2005; Hoff, 2017). On this basis, it has been proposed, and is now widely accepted, that the categorical classification system alone is too reductionist to explain the complexity of psychotic phenomena (Van Os *et al*., 1999; Linscott and van Os, 2010). Various evidence-based perspectives might support a scheme incorporating symptom dimensions in psychotic disorders, as a possible approach to address the following limitations of categorical distinctions.

First, the dichotomous model of non-affective and affective psychosis does not fit the cases presenting with both prominent mood and psychotic symptoms. This is testified by the notion of a third category of schizoaffective disorder (Kasanin, 1933), which nevertheless implies further nosological challenges (Abrams *et al*., 2008).

In addition, if criteria-based classification systems could identify genuine disorders within the psychosis spectrum, the diagnostic overlap would be relevant to only a few patients. On the contrary, there is a large comorbidity index between schizophrenia, schizoaffective, bipolar, and major depressive disorders (Laursen *et al*., 2009; Upthegrove *et al*., 2017). Similarly, the 10-year outcomes of the Aetiology and Ethnicity in Schizophrenia and Other Psychoses (ÆSOP-10) study showed that diagnoses within psychosis other than schizophrenia at baseline tend to be unstable over time (Heslin *et al*., 2015).

Also, the dichotomous model is neither consistent with family studies showing familial co-aggregation of non-affective and affective psychosis (Cardno *et al*., 2002; Lichtenstein *et al*., 2009; Chou *et al*., 2017) nor with the accumulated evidence from genome-wide association studies that genetic risk is in part shared among schizophrenia, bipolar disorder, and major depressive disorder (International Schizophrenia Consortium *et al*., 2009; Demjaha *et al*., 2011; Cardno and Owen, 2014; O'Donovan and Owen, 2016; Power *et al*., 2017).

Last, several studies show the efficacy of agents which impact on dopamine signalling in the treatment of both non-affective and affective symptoms. For example, antipsychotics antagonise D2-receptor functioning and are used in bipolar disorder and schizophrenia (Post, 1999; Taylor *et al*., 2015), and clozapine is prescribed for both treatment-resistant bipolar disorder and schizophrenia (Li *et al*., 2015; Goodwin *et al*., 2016; Howes *et al*., 2016). These findings suggest that dopamine dysregulation may contribute to both positive and manic symptoms, as supported by recent positron emission tomographic findings (Jauhar *et al*., 2017).

Taken together, the above evidence challenges the binary categorization of non-affective and affective psychosis, enhancing research into non-categorical approaches. Pioneering studies using factor analysis examined associations among non-affective symptoms in schizophrenia and showed that these symptoms segregated in three groups (Liddle, 1987); however, these groups could not accommodate the whole symptom diversity in schizophrenia (Kay and Sevy, 1990). Thus, psychopathology models including also depressive and manic factors were proposed and replicated in schizophrenia (Lindenmayer *et al*., 1994; Salokangas, 1997; Wickham *et al*., 2001; Wallwork *et al*., 2012). This type of structure was likewise confirmed in psychotic disorders (Salokangas, 2003; Dikeos *et al*., 2006; Demjaha *et al*., 2009), and in a sample of bipolar patients (Lindenmayer *et al*., 2008). Hence, its validity across the spectrum of non-affective and affective psychosis has been consistently supported.

Recent findings suggest a more fundamental general, transdiagnostic dimension encompassing non-affective and affective symptoms, in addition to five specific symptom dimensions (Reininghaus *et al*., 2013; Reininghaus *et al*., 2016; Shevlin *et al*., 2017). This conceptualization statistically reflects a bifactor model, with one general factor representing shared variance among all symptoms, and a set of specific factors where the remainder of the variance is shared among subsets of symptoms (Reise *et al*., 2007). This is the first study set to investigate, in an incidence sample of First Episode Psychosis (FEP) patients: (1) whether the general psychosis dimension holds across diagnostic categories of non-affective psychosis (i.e. schizophrenia, schizoaffective disorder) and affective psychosis (i.e. bipolar and major depressive disorder with psychotic features); (2) whether formation of specific symptom dimensions is justified in addition to a general psychosis dimension; and (3) the association of demographic characteristics (i.e. age, gender, ethnicity), social context (i.e. urbanicity), and clinical factors (i.e. diagnosis) with general and specific psychosis dimensions.

The hypotheses underlying the third aim, based on the existing literature, were:

(a) Positive symptoms would be more common in ethnic minority groups and in people living in more densely populated environments (van Os *et al*., 2001, Janssen *et al*., 2003).
(b) Negative symptoms would be associated with indices suggestive of neurodevelopment impairment in psychosis (Limosin, 2014; Patel *et al*., 2015), such as being a man or having an early age at onset.

## Methods

### Sample design and procedures

Individuals suffering from their FEP were recruited between 2010 and 2015 as part of the large EUropean network of national schizophrenia networks studying Gene–Environment Interactions (EU-GEI) study (http://www.eu-gei.eu). Specifically, FEP individuals were recruited as part of the ‘Functional Enviromics’ work package, which consisted of an incidence and a case-sibling-control study conducted across six countries with the aim to investigate clinical, genetic, and environmental interaction in the development of psychotic disorders.

The study had 17 catchment areas, including urban and less urban populations: Southeast London, Cambridgeshire and Peterborough (England); central Amsterdam, Gouda and Voorhout (the Netherlands); part of the Veneto region, Bologna municipality, city of Palermo (Italy); 20th arrondissement of Paris, Val-de-Marne, Puy-de-Dôme (France); Madrid (Vallecas), Barcelona, Valencia, Oviedo, Santiago, Cuenca (Spain); and Ribeirão Preto (Brazil).

### Participants

We screened all subjects who were referred to mental healthcare services with a suspicion of psychosis. The ascertainment period of cases ranged from 12 months in London to 48 months in Val-de-Marne and Bologna, with a median of 25 months. In each site, a psychiatrist experienced in epidemiology research oversaw the local team, which was centrally trained to minimize non-differential recruitment bias in the different healthcare systems. Written consent was obtained from the subjects who agreed to take part of the case-sibling-control study. For incidence-only cases, local research ethics committees approved the extraction of demographics and clinical information from patient records. More detailed information is available on the EU-GEI core paper on the incidence rates of schizophrenia and other psychotic disorders (Jongsma *et al*., 2018).

Patients were included in the current study if they met the following criteria during the recruitment period: (a) aged between 18 and 64 years; (b) presentation with a clinical diagnosis for an untreated FEP, even if longstanding [International Statistical Classification of Diseases and Related Health Problems, Tenth Revision (ICD-10) codes F20-F33]; (c) resident within the catchment area at FEP. Exclusion criteria were: (a) previous contact with psychiatric services for psychosis; (b) psychotic symptoms with any evidence of organic causation; and (c) transient psychotic symptoms resulting from acute intoxication (ICD-10: F1*x*.5).

### Measures

Data on age, gender, and ethnicity was collected using a modified version of the Medical Research Council Sociodemographic Schedule (Mallett, 1997). Ethnicity was defined as self-reported. Country of heritage or birth was used as a proxy for ethnicity in people of a North African background. The OPerational CRITeria (OPCRIT) system (McGuffin *et al*., 1991; Williams *et al*., 1996) was used by centrally trained investigators, whose reliability was assessed throughout the study (*κ* = 0.7). The OPCRIT system allows to: (1) assess the pre-morbid history and current mental state; and (2) establish the diagnosis of psychotic disorders based on algorithms for several diagnostic classification systems. It consists of a checklist which can be filled using different sources, e.g. case records or clinical interviews. Fifty-nine items relate to the mental state examination. We used diagnoses based on Research Diagnostic Criteria (RDC) (Spitzer *et al*., 1978), since this classification system provides a better representation of schizoaffective disorder, which is a common presentation in clinical practice. OPCRIT RDC-based diagnoses have a good-to-excellent agreement with best-estimate consensus diagnostic procedures (Craddock *et al*., 1996). In each catchment area, population density was computed as a number of inhabitants per square kilometre, based on official population estimates.

### Statistical analysis

Psychopathology items were dichotomized as 0 ‘absent’ or 1 ‘present’. In order to ensure sufficient covariance coverage for item response modelling, we used the items with a valid frequency of ‘present’ ⩾10% in our sample, which included individuals with ⩽20 missing values in the psychopathology rating. OPCRIT data used in the analysis contained missing values, which we assumed to be missing at random, allowing for the maximum likelihood estimator to provide unbiased estimates. We performed multidimensional item response modelling in M*plus*, version 7.4 (Muthén and Muthén, 2012) to estimate unidimensional, multidimensional, bifactor, and second-order models of psychosis.

Extending previous analyses of OPCRIT data in individuals with enduring psychosis (Reininghaus *et al*., 2016), we estimated five alternative item-response models (online Supplementary Fig. S1): (a) a unidimensional model with one unique general factor (model A), which is consistent with the pre-Kraepelinian unitary concept of psychosis (Berrios and Beer, 1994); (b) a multidimensional model with five uncorrelated specific factors of positive, negative, disorganization, manic, and depressive symptoms (model B); (c) a multidimensional model with five correlated specific factors (model C), which, together with model B, is consistent with the pentagonal psychosis model (van Os and Kapur, 2009); (d) a bifactor model with one general latent factor along with five uncorrelated specific factors (model D) (Reininghaus *et al*., 2016); and (e) a hierarchical model with five first-order specific factors and one general second-order factor (model E), which, as model D, is consistent with the notion of a transdiagnotic spectrum of non-affective and affective psychosis (Craddock and Owen, 2005; Reininghaus *et al*., 2016). Some previous OPCRIT exploratory analysis showed a combined negative/disorganization dimension (Serretti *et al*., 2001; Fanous *et al*., 2005). We did not have a strong theoretical rationale for testing such a structure in a confirmatory analysis. By contrast, we considered specific negative symptoms as a clinically observable marker of neurodevelopmental impairment in psychosis (Limosin, 2014).

The five models were compared using Log-Likelihood (LL), Akaike Information Criterion (AIC), Bayesian Information Criterion (BIC), and Sample-size Adjusted BIC (SABIC) as model fit statistics. For the model showing the best fit, we calculated reliability and strength indices, such as McDonald's omega (*ω*), omega hierarchical (*ω*_H_), and index *H*. Coefficient *ω* is an estimate of the proportion of common variance accounted by general and specific symptom dimensions. Coefficient *ω*_H_ is an estimate of the proportion of reliable variance accounted by the general dimension, treating variability in scores due to specific dimensions as measurement error (Rodriguez *et al*., 2016*b*). Ωh formula can be extended to each specific factor, i.e. treating variability in scores due to the general factor as a measurement error, to compute omega hierarchical for subscales. Based on omega and omega hierarchical coefficients, which can vary from 0 to 1, we computed the ratios of *ω*/*ω*_H_, namely the relative omega, as the amount of reliable variance explained in the observed scores attributable to (1) the general factor independently from the specific symptom dimensions, and (2) each specific symptom dimension independently from the general factor. To estimate the extent to which symptom dimensions were represented by their own set of OPCRIT items and their replicability across studies, we computed the construct reliability index *H* (Hancock and Mueller, 2001). The index *H* ranges from 0 to 1, with values closer to 1 indicating better reliability and replicability (Rodriguez *et al*., 2016*a*). Quantitative scores for all symptom dimensions were calculated using the ‘FSCORES’ function in M*plus*.

Further, we examined the diagnostic classification accuracy based on general and specific symptom dimension scores using multinomial receiver operating characteristic (ROC) analysis in STATA 14 (StataCorp, 2015). In addition, we performed a sensitivity analysis, examining subjects with item ratings based on face-to-face interview and based on clinical records separately.

We used multiple linear regression to examine the association between factor scores of general and/or specific psychosis dimensions as the outcome variable and demographic variables, including gender, age-at-first-contact with psychiatric services, ethnicity, and diagnosis as covariates. Country and assessment method were treated as a priori confounders.

To examine the individual-level effect of urbanicity on symptom dimension scores, standardized population density values were used as a continuous independent variable, while controlling the analysis for gender, age-at-first-contact, ethnicity, diagnosis, and assessment method. Sensitivity analysis included post-hoc multiple regressions within each country, where population density was dichotomized at its median as a dummy variable for urbanicity.

## Results

### Sample characteristics

We identified 2774 treated incidence cases of psychosis (Jongsma *et al*., 2018), of whom 2182 had (complete or missing at random) OPCRIT data available for analysis under the provision of local research ethics committees (Table 1). OPCRIT item ratings were completed based on face-to-face assessment for 51% (*n* = 1112) and based on clinical records for 49% (*n* = 1070) of the sample. The sample prevalence of psychotic symptoms is presented in Supplementary Table S1.

**Table 1.** Demographic and clinical characteristics of the sample included in the factor analysis

Characteristics	*N* (%) 2182	Differences by assessed method^a^ Test statistics	Differences by country^b^ Test statistics
Age			
Mean (s.d.)	32.1 (11.2)	*t*(2180) = −5.57; *p* < 0.001	*F*(5,2176) = 7.42; *p* < 0.001
Median (IQR)	30 (23–40)	Kruskal–Wallis χ^2^(1) = 29.19; *p* < 0.001	Kruskal–Wallis χ^2^(5) = 37.4; *p* < 0.001
Gender^c^			
Male	1247 (57.2)	χ^2^(1) = 14.73; *p* < 0.001	χ^2^(5) = 16.59; *p* < 0.01
Ethnicity^d^			
White	1245 (57.1)	χ^2^(4) = 69.06; *p* < 0.001	χ^2^(20) = 535.15; *p* < 0.001
Black	231 (10.6)		
Mixed	168 (7.7)		
Asian	79 (3.6)		
North African	61 (2.8)		
Other and missing self-reported	398 (18.2)		
Research Domain Criteria Diagnosis^e^			
Bipolar disorder	129 (5.9)	χ^2^(4) = 19.25; *p* = 0.001	χ^2^(20) = 137.47; *p* < 0.001
Major depression with psychotic features	92 (4.2)		
Schizophrenia spectrum	842 (38.6)		
Schizoaffective disorder	764 (35)		
Unspecified psychosis	355 (16.3)		

a Psychopathology assessment methods included face-to-face interview or review of clinical notes.

b Study countries were England, the Netherlands, France, Spain, Italy, and Brazil.

c 29 missing values excluded from tabulation and age analysis.

d Other and missing self-reported groups excluded from ethnicity analysis.

e Schizophrenia spectrum encompassed Broad Schizophrenia (*N* = 194) and Narrow Schizophrenia (*N* = 648); Schizoaffective disorder encompassed Schizoaffective/manic (*N* = 112); Schizoaffective/depressive (*N* = 566); Schizoaffective/bipolar (*N* = 86).

Fifty-seven per cent of FEP were men. Subjects were mostly people of a White ethnicity. Other main ethnic groups included Black African and Black Caribbean, North African, Mixed, and Asian. Mean age-at-first-contact with psychiatric services was 32.1 years; this was lower in men (*M* = 30.1) compared with women (*M* = 34.7; *t* = −9.6, *p* < 0.001). Age-at-first-contact differed across ethnic groups, with individuals of Black ethnicity (*M* = 29) being younger than individuals of White ethnicity (*M* = 32.7; *F* = 7.72, *p* < 0.001). The most common RDC-based diagnosis was broad or narrow schizophrenia (38.6%), followed by schizoaffective disorders (35%), unspecified non-organic psychotic disorder (16.3%), bipolar disorder (5.9%), and psychotic depression (4.2%).

### Symptom dimensions in the EU-GEI sample

The bifactor model was the best fit for the OPCRIT symptom data compared with all other models, as consistently indicated by each of the model fit statistics (Table 2), and explained 54% of the total variance.

**Table 2.** Model fit statistics of unidimensional, multidimensional, bi-factor, and second-order models

Sample size: 2182	Full information fit statistics^a^			
	LL	AIC	BIC	SABIC
A – Unidimensional Model	−54809	109813	110370	110059
B – Multidimensional Model (five uncorrelated factors)	−50645	101487	102044	101733
C – Multidimensional Model (five correlated factors)	−50439	101095	101709	101365
D – Bifactor Model (one general factor and five specific uncorrelated factors)	−49710	99713	100549	100082
E – Hierarchical Model (five first-order specific correlated factors and one second-order general factor)	−50608	101420	102000	101676

LL, log-likelihood; AIC, Akaike Information Criterion; BIC, Bayesian Information Criterion; SABIC Sample-size Adjusted Bayesian Information Criterion.

a A difference of 10 in AIC, BIC and SABIC is considered important. Lower values indicate a statistically better model fit.

**Fig. 1.**
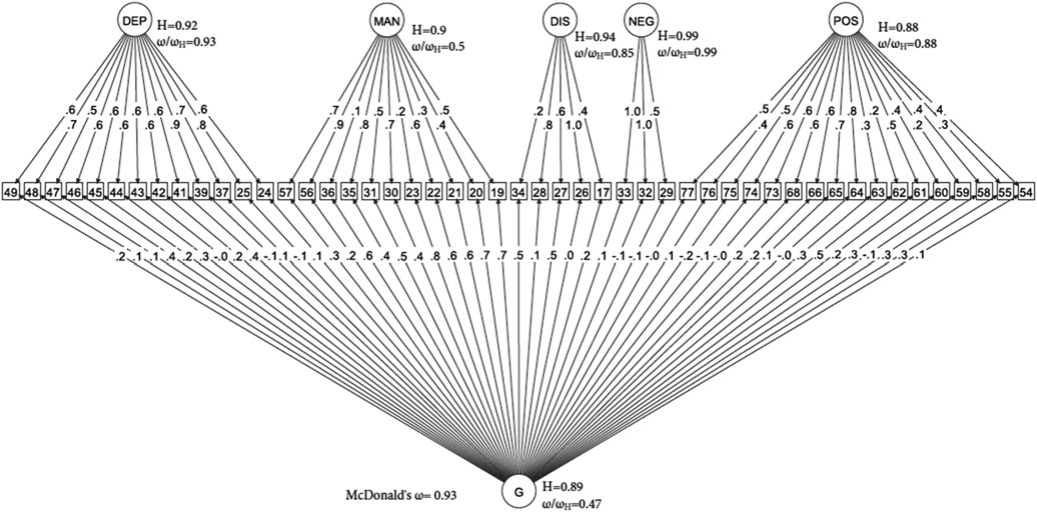
Bifactor model. (□) Observed variables (No. of OPCRIT items); (○) Unobserved variables (latent factors); (→) standardized item loading estimation onto latent factors; G, general psychosis factor; specific symptom factors: DEP, depression; MAN, mania; DIS, disorganization; NEG, negative; POS, positive. Reliability and strength estimates: *H* = construct reliability index; *ω* = McDonald omega; *ω*_H_ = hierarchical omega; *ω*/*ω*_H_ = Relative omega. Explanatory note: McDonald's *ω* is an estimate of the proportion of the common variance accounted by general and specific symptom dimensions. Relative omega (*ω*/*ω*_h_) is the amount of reliable variance explained in the observed scores attributable to (1) the general factor independently from the specific symptom dimensions, and (2) each specific symptom dimension independently from the general factor. *H* is an index of the quality of the measurement model based on the set of OPCRIT items for each symptom dimension. Index *H* can range from 0 to 1, with values closer to 1 indicating a better construct reliability and replicability across studies.

Figure 1 shows that, within the bifactor model, general and specific dimensions accounted for 93% of the common variance. Overall, statistical indices derived from the bifactor model suggest that its explained variance was due to individual differences in both general and specific symptom dimensions, which therefore might complement each other in reflecting the psychopathological structure at FEP. This is illustrated by the relative omega coefficients, which, for example, showed that 47% of the reliable variance was due to the general factor when partitioning out the variability in scores due to the specific factors (Fig. 1). High *H* values were consistently observed for all latent factors, indicating that they were well defined, and that the bifactor model had high reliability and replicability (Fig. 1). Sensitivity analysis showed that the bifactor model was the best fit for the OPCRIT data in both the assessment methods (online Supplementary Tables S2.1 and S2.2).

### Symptom dimensions and item factor loadings

Table 3 shows standardized factor loadings for the bifactor model. On the general dimension, a positive factor loading was observed for all OPCRIT items with statistically significant loadings. In addition, the magnitude of factor loadings of items on the general dimension was small, except for some manic/delusional items for which loadings of moderate magnitude were observed. On the specific dimensions, most of the items showed moderate to strong positive loadings. Finally, latent factor scores were strongly and positively associated with simplified weighted OPCRIT sum scores for use in clinical practice (online Supplementary Table S3).

**Table 3.** Standardized factor loadings in the bifactor model

OPCRIT item	Item no.	Factor	Specific factor loading	General factor loading	Communalities
Persecutory delusions	54	POS	0.36***		0.14
Well organized delusions	55	POS	0.27***	0.34***	0.19
Delusions of influence	58	POS	0.43***	0.33***	0.29
Bizarre delusions	59	POS	0.21***		0.05
Widespread delusions	60	POS	0.42***	0.29***	0.26
Delusions of passivity	61	POS	0.49***		0.27
Primary delusional perception	62	POS	0.23***	0.51***	0.32
Other primary delusions	63	POS	0.30***	0.31***	0.19
Delusions & hallucinations last for 1 week	64	POS	0.81***		0.65
Persecutory/jealous delusions & hallucinations	65	POS	0.66***		0.45
Thought insertion	66	POS	0.60***		0.38
Thought broadcast	68	POS	0.60***	0.24***	0.41
Third person auditory hallucinations	73	POS	0.61***		0.37
Running commentary voices	74	POS	0.62***		0.39
Abusive/accusatory/persecutory voices	75	POS	0.54***		0.33
Other (non-affective) auditory hallucinations	76	POS	0.42***		0.19
Non-affective hallucinations in any modality	77	POS	0.51***		0.26
Negative formal thought disorder	29	NEG	0.54***		0.30
Restricted affect	32	NEG	1.00***		1.00
Blunted affect	33	NEG	0.98***		0.97
Bizarre behaviour	17	DIS	0.42***	0.21***	0.23
Speech difficult to understand	26	DIS	0.96***		0.93
Incoherent	27	DIS	0.62***	0.47***	0.60
Positive formal thought disorder	28	DIS	0.84***		0.72
Inappropriate affect	34	DIS	0.23***	0.46***	0.27
Excessive activity	19	MAN	0.53***	0.73***	0.82
Reckless activity	20	MAN	0.36***	0.67***	0.58
Distractibility	21	MAN	0.29***	0.60***	0.45
Reduced need for sleep	22	MAN	0.55***	0.56***	0.61
Agitated activity	23	MAN	0.16***	0.76***	0.59
Pressured speech	30	MAN	0.74***	0.43***	0.73
Thoughts racing	31	MAN	0.54***	0.49***	0.53
Elevated mood	35	MAN	0.85***	0.41***	0.89
Irritable mood	36	MAN	0.12**	0.55***	0.32
Increased self esteem	56	MAN	0.87***	0.24***	0.81
Grandiose delusions	57	MAN	0.67***	0.30***	0.54
Slowed activity	24	DEP	0.55***		0.31
Loss of energy/tiredness	25	DEP	0.80***		0.64
Dysphoria	37	DEP	0.74***		0.55
Loss of pleasure	39	DEP	0.87***		0.76
Poor concentration	41	DEP	0.62***	0.42***	0.56
Excessive self-reproach	42	DEP	0.60***		0.38
Suicidal ideation	43	DEP	0.55***		0.31
Initial insomnia	44	DEP	0.65***	0.32***	0.53
Middle insomnia (broken sleep)	45	DEP	0.65***	0.25***	0.48
Early morning waking	46	DEP	0.56***	0.39***	0.46
Excessive sleep	47	DEP	0.46***		0.23
Poor appetite	48	DEP	0.69***		0.48
Weight Loss	49	DEP	0.56***	0.20***	0.35

General, general psychosis factor; specific symptom dimensions: DEP, depression; MAN, mania; DIS, disorganisation; NEG, negative; POS, positive. Only loadings ⩾0.2 for the general factor are shown for simplicity. Significance: *** = *p* < 0.001; ** = *p* < 0.01.

### Symptom dimensions and categorical diagnoses

**Fig. 2.**
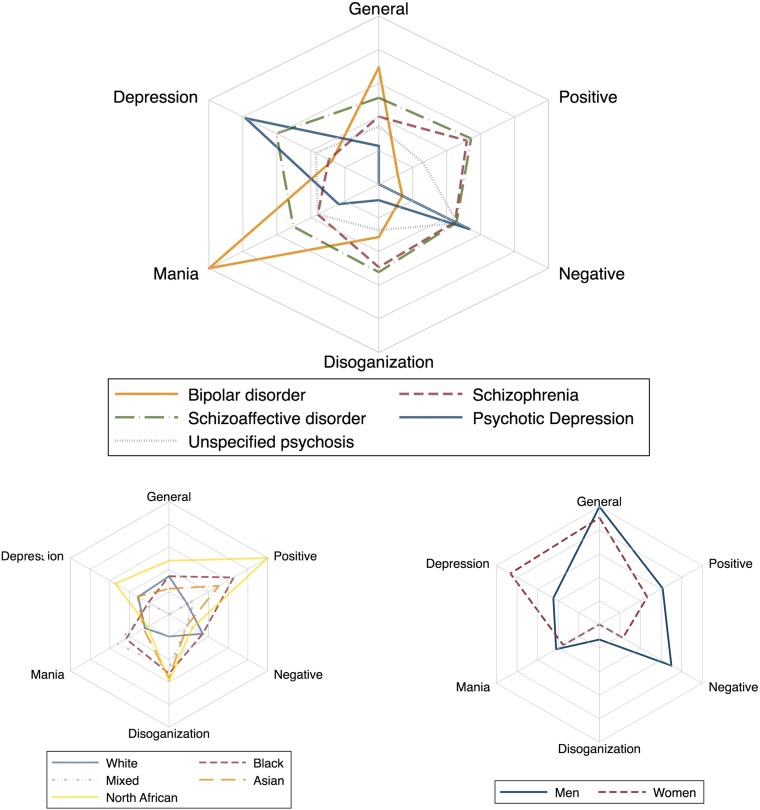
Predicted symptom profiles by RDC-based diagnostic category, gender, and ethnicity. Explanatory note: After the estimation of the bifactor model, the continuous scores for general and specific symptom dimensions were computed using the function ‘FSCORES’ in *Mplus* (setting mean = 0 and standard deviation = 1), and used as the outcome variable in the regression analyses.

Findings from regression analyses are shown in Table 4 and predicted symptom dimension scores for each RDC-based diagnostic category are reported in Fig. 2. Compared with bipolar disorder, factor scores for the positive dimension were moderately higher in schizophrenia and schizoaffective disorder; factor scores for the negative dimension were moderately higher in schizophrenia, schizoaffective and psychotic depression; and factor scores for the depressive dimension were markedly higher in psychotic depression and schizoaffective disorder. Bipolar disorder showed the highest factor scores for the manic and the general dimensions. Dimension scores based on ICD diagnostic categories are presented in Supplementary Fig. S2 and Supplementary Table S4.

**Table 4.** Symptom dimension scores by sociodemographic, categorical diagnosis, and social context variables

	General B (95% CI)	Positive B (95% CI)	Negative B (95% CI)	Disorganization B (95% CI)	Manic B (95% CI)	Depressive B (95% CI)
Women *v*. Men^a^	0.01 (−0.07 to 0.09)	0.01 (−0.08 to 0.1)	−0.12** (−0.21 to 0.23)	0 (−0.08 to 0.1)	0 (−0.09 to 0.08)	0.1** (0.02 to 0.17)
Age at first contact^a^	−0.01* (−0.09 to −0.01)	−0.02 (−0.06 to 0.03)	−0.05** (−0.1 to −0.01)	−0.09*** (−0.14 to −0.05)	−0.1*** (−0.14 to −0.06)	0.04* (0.01 to 0.08)
Ethnicity^a^						
Black *v*. White	0.07 (−0.06 to 0.19)	0.19** (0.04 to 0.33)	0.01 (−0.14 to 0.15)	0.14* (0.01 to 0.28)	0.03 (−0.1 to 0.16)	−0.22*** (−0.34 to −0.1)
Mixed *v*. White	0.02 (−0.12 to 0.17)	0 (−0.16 to 0.17)	0.1 (−0.07 to 0.27)	0.18* (0.02 to 0.34)	0.06 (−0.09 to 0.21)	−0.1 (−0.25 to 0.03)
Asian *v*. Withe	−0.06 (−0.25 to 0.13)	0.11 (−0.1 to 0.33)	−0.05 (−0.28 to 0.18)	0.07 (−0.13 to 0.28)	0.01 (−0.19 to 0.21)	−0.08 (−0.27 to 0.1)
North African *v*. White	−0.02 (−0.24 to 0.2)	0.32** (0.07 to 0.57)	−0.22 (−0.48 to 0.04)	−0.05 (−0.29 to 0.2)	−0.17 (−0.4 to 0.06)	0.05 (−0.16 to 0.27)
Diagnosis^a^						
Schizophrenia *v*. Bipolar	−0.78*** (−0.96 to −0.6)	0.9*** (0.69 to 1.1)	0.53*** (0.32 to 0.75)	0.24* (0.06 to 0.45)	−1.7*** (−1.88 to −1.51)	0.78 (−0.1 to 0.25)
Schizoaffective disorder *v*. Bipolar	−0.47*** (−0.65 to −0.29)	0.94*** (0.73 to 1.14)	0.59*** (0.37 to 0.8)	0.3** (0.1 to 0.5)	−1.33*** (−1.52 to −1.15)	0.97*** (0.8 to 1.14)
Major Depression *v*. Bipolar	−1.16*** (−1.42 to −0.91)	−0.24 (−0.52 to 0.05)	0.72*** (0.42 to 1.02)	−0.23 (−0.5 to 0.05)	−1.95*** (−2.21 to −1.69)	1.54*** (1.3 to 1.79)
Unspecified Functional Psychosis *v*. Bipolar	−0.99*** (−1.19 to −0.8)	0.36** (0.14 to 0.58)	0.5*** (0.27 to 0.73)	−0.06 (−0.27 to 0.15)	−1.67*** (−1.87 to −1.47)	0.3** (0.11 to 0.49)
Urban *v*. less urban^b^	0.3*** (0.24 to 0.36)	−0.03 (−0.1 to 0.03)	0.12** (0.05 to 0.19)	0.08** (−0.02 to 0.14)	0.01 (−0.06 to 0.06)	0.02 (−0.04 to 0.07)

B, unstandardised regression coefficient; CI, confidence interval.

a Covariates in multiple models were gender, age, ethnicity, diagnosis, study country, and type of assessment method (interview *v*. case records).

b Population density analysis was adjusted for gender, age, ethnicity, diagnosis, and type of assessment method (interview *v*. case records).

Finally, ROC analysis showed that classification accuracy into RDC categories based on general and specific symptom dimension scores was markedly higher for patients with psychopathology rating based either on face-to-face interview (95% CI 0.54–0.63) or case note review (95% CI 0.56–0.65), compared with a classification by chance (95% 0.32–0.41). Moreover, symptom dimensions showed similar diagnostic classification accuracy across countries (online Supplementary Figs S3.1 and S3.2).

### Symptom dimensions by gender, age-at-first-contact, and ethnicity

Findings on factor scores by gender, age-at-first-contact, and ethnicity, are shown in Fig. 2 and Table 4. Early age-at-first-contact was associated with higher scores for the general, negative, disorganized, and manic symptom dimensions, and with lower scores for the depressive symptom dimension. Men showed fewer depressive symptoms and more negative symptoms than women, even after adjusting the analysis for several confounders. Table 4 further shows that participants of Black and North African ethnicity presented with higher scores on the positive symptom dimension compared with an individual of White ethnicity. Finally, higher scores for the disorganization dimension and lower scores for the depressive dimension were observed in Black compared with White ethnicity. Noteworthy, the magnitude of the effect was small for all the results.

### Symptom dimensions by urbanicity

A moderate positive association was observed for more densely populated environments and the general dimension score. Table 4 further shows a weaker positive association between population density and specific negative, disorganization, and manic symptom dimensions. Post-hoc analysis of symptom dimensions within countries showed that positive symptoms were more common in urban study sites in the UK (i.e. London *v.* Cambridge), whereas a negative association was observed in Spain (online Supplementary Table S5).

## Discussion

### Principal findings

This is the first study on general and specific symptom dimensions in an incidence sample of psychosis. First, we found in our FEP sample that manic and delusional symptoms primarily underlie the identified general psychosis factor across diagnostic categories of non-affective and affective psychosis. Second, findings showed that specific dimensions of positive, negative, disorganized, manic and depressive symptoms are complementary to the general dimension. Third, general and specific symptom dimensions discriminated well between diagnoses of psychotic disorders. Forth, positive symptoms were more common among individuals of Black and North African ethnicity. Fifth, there was some evidence that early age-at-first-contact was associated with higher scores for several dimensions, such as of negative, disorganised and manic symptoms. Sixth, men presented with more negative and less depressive symptoms than women. Finally, higher scores for the general dimension were observed for individuals living in urban neighbourhoods.

### Limitations

Before interpreting our findings, we must consider potential limitations. Symptoms were rated with a semi-structured face-to-face interview or from case note review. Still, study investigators underwent a specific and centrally organized training for OPCRIT and demonstrated good inter-rater reliability for individual item ratings; moreover, OPCRIT is a tool specifically designed to allow use with different sources (McGuffin *et al*., 1991; Cardno *et al*., 1996; Rucker *et al*., 2011). However, we found consistently lower symptom ratings using case note review compared with face-to-face interviews. It is possible that clinicians failed to record all symptoms; alternatively, patients presenting with less severe psychopathology had a shorter contact with services, and therefore less chances to be interviewed by researchers. Whether or not differences in ratings are genuine or a surrogate of different sources of item ratings, we treated this potential bias as artificial confounding of our findings and adjusted all analyses for the type of assessment method. On the other hand, the use of an incidence sample allowed the best possible approximation of the true distribution of psychosis symptoms at FEP, which may have reduced potentially inflated presence of positive and negative symptoms in previous studies conducted in hospital settings (Allardyce *et al*., 2007). Also, OPCRIT does not cover some relevant aspects of negative symptoms related to passive social withdrawal, lack of motivation, and difficulties in abstract/symbolic thinking. Consequently, we constructed a narrow negative symptom dimension with three items. Finally, some authors have argued that, in a bifactor model, the general factor may be difficult to interpret and in general may overfit the data (Bonifay *et al*., 2016). However, the bifactor model allows solutions to dimensionality issues that arise when the conceptual breadth of a construct cannot be fully determined (Reise *et al*., 2007), as is likely to be the case for the construct of psychosis, which, in the past, has been considered as unidimensional and multidimensional at the same time. For example, the bifactor model discerns each specific symptom dimension from the common item effect, which is captured by the general dimension, thus allowing an accurate evaluation of the unique contribution of each subset of symptoms. Last, this solution provides crucial information which cannot be determined from the other models, i.e. how much of the phenotypic variance that we aim to measure is due to a unidimensional construct *v.* a multidimensional construct of psychosis. Hence, it was a suitable model for addressing dimensionality issues for psychosis and generating reliable phenotypes.

### Comparison with previous research

In our study, the bifactor model of psychopathology best explained the observed symptoms at FEP compared with unidimensional and multidimensional models. Our findings are consistent with, and extend, previous research on psychotic symptoms in people with enduring psychotic disorders (Reininghaus *et al*., 2013; Reininghaus *et al*., 2016) and the general population (Shevlin *et al*., 2017) to a multinational incidence sample of FEP. They provide further evidence that non-affective and affective psychotic disorders lie on a common mood-psychosis spectrum (Murray *et al*., 2004). In addition, we provided the first evidence in psychosis that a bifactor solution shows better model fit statistics compared with a second-order hierarchical solution. However, compared with findings in enduring psychosis (Reininghaus *et al*., 2016), we found a less specific general psychopathology factor with more general disturbances and affective features. As illnesses develop, the non-affective psychotic phenomena may become more and affective features less prominent.

We found some evidence of gender differences in symptom dimension scores. Men showed less depressive symptoms and more negative symptoms compared with women. This finding is consistent with other studies in stable schizophrenia (Shtasel *et al*., 1992; Roy *et al*., 2001; Galderisi *et al*., 2012), first episode psychotic disorder (Morgan *et al*., 2008), and the general population (Maric *et al*., 2003). In our sample, we also showed that early age-at-first-contact was associated with a higher level of general and specific psychopathology. Notably, it has been proposed that gender-related and symptom profiles differences in psychosis may be suggestive of different neurodevelopmental trajectories (Castle and Murray, 1991; Seeman, 1997; Riecher-Rössler and Häfner, 2000).

We further found that symptom dimensions vary in terms of ethnicity. Consistent with a previous report (Kirkbride *et al*., 2016), we provided evidence that people of Black ethnicity presented at FEP with more positive and disorganized symptoms and fewer depressive symptoms compared with people of White ethnicity. Moreover, in line with another study (Veling *et al*., 2007), we found in our sample that the North African group presented at FEP with more positive symptoms compared with people of White ethnicity. It has been debated whether similar findings reflect true differences in symptom presentation or instead result from raters being more likely to overrate symptoms in the context of ethno-cultural diversity (Mukherjee *et al*., 1983; Hutchinson *et al*., 1999; Barrio *et al*., 2003; Arnold *et al*., 2004; Vega and Lewis-Fernandez, 2008). Recent studies using standardized procedures for assessing symptomatology blind to ethnicity have suggested that misdiagnosis or rating bias cannot account for differences across ethnic groups (Morgan *et al*., 2010). However, we must remain cautious in interpreting these results.

We showed that high population density is positively associated with the general and specific disorganized, negative and manic dimensions. In our multinational sample, we were not able to replicate previous findings on the relationship between urbanicity and the positive dimension (Kirkbride *et al*., 2007). Nevertheless, stratified analysis by country was consistent with the previously reported association between urbanicity and positive symptoms in the UK. The relationship between urbanicity and a higher incidence of psychotic disorders is well-established (Vassos *et al*., 2012). However, it has been found to show non-linearity (Kirkbride *et al*., 2017), which implies that the effect of urbanicity may depend on exposure to additional socio-environmental factors associated with urban contexts, for example cannabis use (Kuepper *et al*., 2011) and childhood adversities (Frissen *et al*., 2015). Similarly, our findings support the hypothesis that urban environment does not have a dimension-specific effect and may act to confer risk for different psychopathological outcomes in psychosis (van Os *et al*., 2002). Noteworthy, similar findings have been reported in the general population (van Os *et al*., 2001), which may require future studies to consider the additive interaction between putative risk factors for psychosis and urbanicity.

## Implications

In the context of a general effort to move away from DSM and ICD categories (Demjaha *et al*., 2009; Reininghaus *et al*., 2016; Kotov *et al*., 2017; Van Dam *et al*., 2017; Whalen, 2017; Zachar and Kendler, 2017), we found evidence that supports, and may inform, the use of dimensional measures in the field of psychosis. In our sample, the bifactor model was a valid platform for research into FEP. Nevertheless, the plausibility of our statistically-guided approach depends on the extent to which: (1) symptom dimensions represent coherent environmental and biological factors; and (2) meaningful clinical information or decisions may derive from the latent constructs.

From a research perspective, our findings suggest that the general dimension may reflect a phenotype for the study of general risk factors. For example, urbanicity may impact on the risk and profile of psychosis through the combination of other, more specific socio- or bio-environmental factors. In addition, we showed a substantial variation of sociodemographic determinants at the specific dimension level, which may support an integrated socio-developmental model of psychosis (Morgan *et al*., 2010).

We may further suggest using the general dimension as a quantitative measure of psychopathology for research into the genetic component shared across psychotic disorders. The evidence is required to establish the extent to which pathophysiology of schizophrenia, bipolar disorder, and psychotic depression is shared at the level of pathways and neuronal cell mechanisms (Forstner *et al*., 2017). Based on the data presented on specific symptom dimensions, it is intriguing to speculate whether the distribution of psychotic symptoms reflects a gradient of neurodevelopmental impairment or socio-environmental risk (Morgan *et al*., 2010; Howes and Murray, 2014) resulting in different patterns of functional abnormalities (Murray and Lewis, 1987; Murray *et al*., 1992; Demjaha *et al*., 2011; Owen and O'Donovan, 2017).

From a clinical perspective, although each patient presents with a specific pattern of psychopathology and response to treatment at FEP, attention has been traditionally focused on the positive dimension management. Mental health professionals may integrate observations of the whole range of symptoms and signs with a consideration of neurodevelopmental and socio-environmental risk factors. Such an approach should aim to plan and optimize pharmacological and non-pharmacological treatments (Murray *et al*., 2016), thus focusing further on treatment of negative, disorganized and affective dimensions (Wykes *et al*., 2011; Giacco *et al*., 2012; Carbon and Correll, 2014; Pelayo-Teran *et al*., 2014; Rosenbaum *et al*., 2014).

We may further suggest promoting mental health professionals to adopt treatment plans guided by dimensions, and increasing their confidence in dimensional classifications. Reconciling contradictory concerns of clinicians and researchers (Kendell and Jablensky, 2003) may represent the first milestone towards a gradual nosology refinement.

## Supplementary material

For supplementary material accompanying this paper visit https://doi.org/10.1017/S0033291718002131. click here to view supplementary material

## Appendix

### Non-author EU-GEI collaborators

Kathryn Hubbard^1^, Stephanie Beards^1^, Simona A. Stilo^2^, Mara Parellada^3^, Pedro Cuadrado^4^, José Juan Rodríguez Solano^5^, Angel Carracedo^6^, Enrique García Bernardo^7^, Laura Roldán^3^, Gonzalo López^3^, Bibiana Cabrera^8^, Esther Lorente-Rovira^9^, Paz Garcia-Portilla^10^, Javier Costas^6^, Estela Jiménez-López^11^, Mario Matteis^3^, Marta Rapado^3^, Emiliano González^3^, Covadonga Martínez^3^, Emilio Sánchez^7^, M^a^ Soledad Olmeda^7^, Nathalie Franke^12^, Fabian Termorshuizen^13,14^, Daniella van Dam^12^, Elsje van der Ven^13,14^, Elles Messchaart^14^, Marion Leboyer^15–18^, Franck Schürhoff^15–18^, Stéphane Jamain^16–18^, Grégoire Baudin^15,16^, Aziz Ferchiou^15,16^, Baptiste Pignon^15,16,18^, Jean-Romain Richard^16,18^, Thomas Charpeaud^18,19,21^, Anne-Marie Tronche^18,19,21^, Flora Frijda^22^, Giovanna Marrazzo^23^, Lucia Sideli^22^, Crocettarachele Sartorio^22,23^, Fabio Seminerio^22^, Camila Marcelino Loureiro^24,25^, Rosana Shuhama^24,25^, Mirella Ruggeri^26^, Sarah Tosato^26^, Chiara Bonetto^26^ and Doriana Cristofalo^26^.

### Affiliations

^1^Department of Health Service and Population Research, Institute of Psychiatry, King's College London, De Crespigny Park, Denmark Hill, London SE5 8AF, UK

^2^Department of Psychosis Studies, Institute of Psychiatry, King's College London, De Crespigny Park, Denmark Hill, London SE5 8AF, UK

^3^Department of Child and Adolescent Psychiatry, Hospital General Universitario Gregorio Marañón, School of Medicine, Universidad Complutense, IiSGM (CIBERSAM), C/Doctor Esquerdo 46, 28007 Madrid, Spain

^4^Villa de Vallecas Mental Health Department, Villa de Vallecas Mental Health Centre, Hospital Universitario Infanta Leonor / Hospital Virgen de la Torre, C/San Claudio 154, 28038 Madrid, Spain

^5^Puente de Vallecas Mental Health Department, Hospital Universitario Infanta Leonor/Hospital Virgen de la Torre, Centro de Salud Mental Puente de Vallecas, C/Peña Gorbea 4, 28018 Madrid, Spain

^6^Fundación Pública Galega de Medicina Xenómica, Hospital Clínico Universitario, Choupana s/n, 15782 Santiago de Compostela, Spain

^7^Department of Psychiatry, Hospital General Universitario Gregorio Marañón, School of Medicine, Universidad Complutense, IiSGM (CIBERSAM), C/Doctor Esquerdo 46, 28007 Madrid, Spain

^8^Department of Psychiatry, Hospital Clinic, IDIBAPS, Centro de Investigación Biomédica en Red de Salud Mental (CIBERSAM), Universidad de Barcelona, C/Villarroel 170, escalera 9, planta 6, 08036 Barcelona, Spain

^9^Department of Psychiatry, School of Medicine, Universidad de Valencia, Centro de Investigación Biomédica en Red de Salud Mental (CIBERSAM), C/Avda. Blasco Ibáñez 15, 46010 Valencia, Spain

^10^Department of Medicine, Psychiatry Area, School of Medicine, Universidad de Oviedo, Centro de Investigación Biomédica en Red de Salud Mental (CIBERSAM), C/Julián Clavería s/n, 33006 Oviedo, Spain

^11^Department of Psychiatry, Servicio de Psiquiatría Hospital “Virgen de la Luz”, C/Hermandad de Donantes de Sangre, 16002 Cuenca, Spain

^12^Department of Psychiatry, Early Psychosis Section, Academic Medical Centre, University of Amsterdam, Meibergdreef 5, 1105 AZ Amsterdam, The Netherlands

^13^Rivierduinen Centre for Mental Health, Leiden, Sandifortdreef 19, 2333 ZZ Leiden, The Netherlands

^14^Department of Psychiatry and Neuropsychology, School for Mental Health and Neuroscience, South Limburg Mental Health Research and Teaching Network, Maastricht University Medical Centre, P.O. Box 616, 6200 MD Maastricht, The Netherlands

^15^AP-HP, Groupe Hospitalier “Mondor”, Pôle de Psychiatrie, 51 Avenue de Maréchal de Lattre de Tassigny, 94010 Créteil, France

^16^INSERM, U955, Equipe 15, 51 Avenue de Maréchal de Lattre de Tassigny, 94010 Créteil, France

^17^Faculté de Médecine, Université Paris-Est, 51 Avenue de Maréchal de Lattre de Tassigny, 94010 Créteil, France

^18^Fondation Fondamental, 40 Rue de Mesly, 94000 Créteil, France

^19^CMP B CHU, BP 69, 63003 Clermont Ferrand, Cedex 1, France

^20^EPS Maison Blanche, Paris 75020, France

^21^Université Clermont Auvergne, EA 7280, Clermont-Ferrand 63000, France

^22^Department of Experimental Biomedicine and Clinical Neuroscience, Section of Psychiatry, University of Palermo, Via G. La Loggia n.1, 90129 Palermo, Italy

^23^Unit of Psychiatry, “P. Giaccone” General Hospital, Via G. La Loggia n.1, 90129 Palermo, Italy

^24^Departamento de Neurociências e Ciencias do Comportamento, Faculdade de Medicina de Ribeirão Preto, Universidade de São Paulo, Av. Bandeirantes, 3900-Monte Alegre- CEP 14049-900, Ribeirão Preto, SP, Brasil

^25^Núcleo de Pesquina em Saúde Mental Populacional, Universidade de São Paulo, Avenida Doutor Arnaldo 455, CEP 01246-903, SP, Brasil

^26^Section of Psychiatry, Department of Neuroscience, Biomedicine and Movement, University of Verona, Piazzale L.A. Scuro 10, 37134 Verona, Italy
