# Supplementary material for: Transdiagnostic dimensions of psychopathology at first episode psychosis: findings from the multinational EU-GEI study
Source: Psychol Med. 2018 Oct 4;49(8):1378–91. doi: 10.1017/S0033291718002131 (PMC6518388; doi:10.1017/S0033291718002131)
Supplement: Supplementary file 1 [file S0033291718002131sup001.docx]

**Supplementary Table S1**

Prevalence of non-affective and affective psychotic symptoms in the analysed sample

| OPCRIT ITEM | Item no. | Factor | Valid frequency  Total sample | Valid frequency  Face-to-face assessment | Valid frequency  Case note review |
| --- | --- | --- | --- | --- | --- |
| Persecutory Delusions | 54 | POS | 71.1% (1,551) | 71.6% (794) | 71% (757) |
| Well organised delusions | 55 | POS | 35.1% (765) | 41.6% (458) | 28.8% (307) |
| Delusions of influence | 58 | POS | 33.3% (726) | 24.1% (267) | 15.4% (165) |
| Bizarre Delusions | 59 | POS | 30.7% (669) | 23.3% (259) | 11.3% (121) |
| Widespread Delusions | 60 | POS | 34.4% (751) | 42.4% (437) | 29.6% (314) |
| Delusions of passivity | 61 | POS | 12.2% (264) | 15.2% (168) | 9% (96) |
| Primary delusional perception | 62 | POS | 20.5% (440) | 26.2% (286) | 14.6% (154) |
| Other primary delusions | 63 | POS | 17% (370) | 19.4% (213) | 14.9% (157) |
| Delusions & hallucinations last for one week | 64 | POS | 51.4% (1076) | 47.9% (495) | 54.8% (581) |
| Persecutory delusions & hallucinations | 65 | POS | 28.1% (591) | 30.1% (311) | 26.2% (280) |
| Thought insertion | 66 | POS | 11.2% (241) | 16.4% (180) | 5.8% (61) |
| Thought broadcast | 68 | POS | 10.2% (221) | 15.5% (171) | 4.7% (50) |
| Third person auditory hallucinations | 73 | POS | 24.5% (531) | 29.3% (322) | 19.7% (209) |
| Running commentary voices | 74 | POS | 19.5% (422) | 24.1% (266) | 14.7% (156) |
| Abusive/accusatory/persecutory voices | 75 | POS | 35% (732) | 31.8% (329) | 38.1% (403) |
| Other (non-affective) auditory hallucinations | 76 | POS | 20.6% (446) | 23.3% (264) | 17.2% (182) |
| Non-affective hallucination in any modality | 77 | POS | 24.8% (537) | 26.7% (294) | 23% (243) |
| Negative formal thought disorder | 29 | NEG | 17.5% (378) | 19% (209) | 16%(169) |
| Restricted affect | 32 | NEG | 31.3% (679) | 36.4% (404) | 25.9% (275) |
| Blunted affect | 33 | NEG | 17.7% (374) | 21.9% (243) | 12.3% (131) |
| Bizarre behaviour | 17 | DIS | 53.1% (1,147) | 44.9% (496) | 61.7% (651) |
| Speech difficult to understand | 26 | DIS | 24% (520) | 20.9% (230) | 27.2% (290) |
| Incoherent | 27 | DIS | 10.4% (226) | 13% (13) | 7.7% (82) |
| Positive formal thought disorder | 28 | DIS | 25.8% (558) | 24.3% (268) | 27.3% (290) |
| Inappropriate affect | 34 | DIS | 16.2% (351) | 19.6% (216) | 12.7% (135) |
| Excessive activity | 19 | MAN | 19.6% (426) | 25.5% (283) | 13.5% (143) |
| Reckless activity | 20 | MAN | 15.2% (330) | 21% (233) | 9.1% (97) |
| Distractibility | 21 | MAN | 37% (799) | 47.4% (521) | 26.3% (278) |
| Reduced need for sleep | 22 | MAN | 26.1% (565) | 30.8% (340) | 21.2% (225) |
| Agitated activity | 23 | MAN | 34.1% (740) | 41.3% (457) | 26.7% (283) |
| Pressured speech | 30 | MAN | 20.3% (440) | 23% (255) | 17.4% (185) |
| Thoughts racing | 31 | MAN | 21.6% (467) | 33% (365) | 9.7% (102) |
| Elevated mood | 35 | MAN | 18.1% (395) | 20.6% (229) | 15.5% (166) |
| Irritable mood | 36 | MAN | 39.4% (857) | 47.7% (529) | 30.7% (328) |
| Increased self esteem | 56 | MAN | 19.8% (432) | 24.1% (267) | 15.4% (165) |
| Grandiose Delusions | 57 | MAN | 17.4% (380) | 23.3% (259) | 11.3% (121) |
| Slowed activity | 24 | DEP | 16.1% (349) | 23.6% (261) | 8.3% (88) |
| Loss of energy/tiredness | 25 | DEP | 33.7% (729) | 40.1% (444) | 26.7% (285) |
| Dysphoria | 37 | DEP | 46.4% (1,009) | 48.7% (540) | 44% (469) |
| Loss of pleasure | 39 | DEP | 37.8% (815) | 43.2% (477) | 32% (338) |
| Poor concentration | 41 | DEP | 49.1% (1,061) | 61% (676) | 36.6% (385) |
| Excessive self-reproach | 42 | DEP | 19.4% (422) | 25.8% (286) | 12.8% (136) |
| Suicidal ideation | 43 | DEP | 27.9% (606) | 34.2% (380) | 21.3% (226) |
| Initial insomnia | 44 | DEP | 46.7% (1,005) | 52.4% (576) | 40.8% (429) |
| Middle insomnia (broken sleep) | 45 | DEP | 33.6% (723) | 38.4% (423) | 28.6% (300) |
| Early morning waking | 46 | DEP | 17.3% (372) | 24.9% (274) | 9.3% (98) |
| Excessive sleep | 47 | DEP | 10.6% (228) | 15.2% (168) | 5.7% (60) |
| Poor appetite | 48 | DEP | 34.6% (743) | 37% (407) | 32.1% (336) |
| Weight Loss | 49 | DEP | 22.1% (469) | 29.3% (315) | 14.8% (154) |

**Supplementary Tables S2.1 and S2.2**

Model fit statistics of unidimensional, multidimensional, bi-factor, and second-order models for different assessment methods

**Supplementary Table S2.1**

Item ratings based on face-to-face interview^a^

| Sample size: 1,112 | | | | |
| --- | --- | --- | --- | --- |
|  | Full information fit statistics^b^ | | | |
|  | LL | AIC | BIC | SABIC |
| A - Unidimensional Model | -29965 | 60126 | 60618 | 60306 |
| B - Multidimensional Model (five uncorrelated factors) | -28070 | 56335 | 56826 | 56515 |
| C - Multidimensional Model (five correlated factors) | -27894 | 56004 | 56546 | 56202 |
| D - Bifactor Model (one general factor and five specific uncorrelated factors) | -27597 | 55489 | 56226 | 55759 |
| E - Hierarchical Model (five first-order specific correlated factors and one second order general factor) | -27995 | 56197 | 56713 | 56386 |

LL, log-likelihood; AIC, Akaike Information Criterion; BIC, Bayesian Information Criterion; SABIC Sample-size Adjusted Bayesian Information Criterion

a. Only items with a valid frequency of ‘present’ ≥10% were analysed

b. A difference of 10 in AIC, BIC and SABIC is considered important. Lower values indicate a statistically better model fit

**Supplementary Table S2.2**

Item ratings based on case note review^a^

| Sample size: 1,070 | | | | |
| --- | --- | --- | --- | --- |
|  | Full information fit statistics^b^ | | | |
|  | LL | AIC | BIC | SABIC |
| A - Unidimensional Model | -23708 | 47595 | 48037 | 47755 |
| B - Multidimensional Model (five uncorrelated factors) | -22239 | 44656 | 45099 | 44816 |
| C - Multidimensional Model (five correlated factors) | -22159 | 44515 | 45008 | 44693 |
| D - Bifactor Model (one general factor and five specific uncorrelated factors) | -21668 | 43594 | 44236 | 43826 |
| E - Hierarchical Model (five first-order specific correlated factors and one second order general factor) | -22227 | 44640 | 45103 | 44808 |

LL, log-likelihood; AIC, Akaike Information Criterion; BIC, Bayesian Information Criterion; SABIC Sample-size Adjusted Bayesian Information Criterion

a. Only items with a valid frequency of ‘present’ ≥10% were analysed

b. A difference of 10 in AIC, BIC and SABIC is considered important. Lower values indicate a statistically better model fit

**Supplementary Table S3**

Correlation between OPCRIT simplified scores and latent factor scores derived from the confirmatory factor analysis**^a^**

| Symptom dimension | r (95% CI)^b^ |
| --- | --- |
| General | 0.84 (0.82 to 0.84) |
| Positive | 0.9 (0.89 to 0.91) |
| Negative | 0.96 (0.96) |
| Disorganization | 0.93 (0.92 to 0.93) |
| Mania | 0.85 (0.84 to 0.86) |
| Depression | 0.96 (0.96 to 0.97) |

a. OPCRIT simplified scores for each symptom dimension were obtained by the sum of the item weighted by the sign of the factor loading and divided by the number of valid items in each observation

b. All p values <0.001

**Supplementary Table S4**

Latent factor scores by ICD-10 diagnosis**^a^**

|  | General  B (95% CI) | Positive  B (95% CI) | Negative  B (95% CI) | Disorganization  B (95% CI) | Mania  B (95% CI) | Depression  B (95% CI) |
| --- | --- | --- | --- | --- | --- | --- |
| **ICD-10 Diagnosis^a^** |  |  |  |  |  |  |
| Schizophrenia  v. Bipolar | -0.86***  (-1 to -0.73) | 0.94***  (0.78 to 1.1) | 0.55***  (0.39 to 0.71) | -0.08  (-0.23 to -0.07) | -1.5***  (-1.63 to -1.36) | 0.18**  (0.05 to 0.31) |
| Schizoaffective disorder  v. Bipolar | -0.3*  (-0.58 to -0.07) | 1.01***  (0.71 to 1.03) | 0.7***  (0.39 to 1.01) | -0.4**  (-0.68 to -0.11) | -1.14***  (-1.4 to -0.88) | 0.91***  (0.66 to 1.17) |
| Major Depression  v. Bipolar | -1.3***  (-1.45 to -1.13) | 0.39***  (0.20 to 0.58) | 0.65***  (0.46 to 0.85) | -0.45***  (-0.63 to -0.26) | -1.7***  (-1.87 to -1.53) | 1.56***  (1.4 to 1.72) |
| Unspecified Functional Psychosis  v. Bipolar | -0.92***  (-1 to -0.78) | 0.37***  (0.22 to 0.53) | 0.49***  (0.33 to 0.65) | -0.22**  (-0.37 to -0.11) | -1.14***  -1.4 to -0.88) | 0.27***  (0.14 to 0.41) |

a. The analyses were controlled for gender, age, ethnicity, country, and type of assessment method (interview v. case records)

**Supplementary Table S5**

Latent factor scores by urbanicity within country^a,b^

|  | General  B (95% CI) | Positive  B (95% CI) | Negative  B (95% CI) | Disorganization  B (95% CI) | Mania  B (95% CI) | Depression  B (95% CI) |
| --- | --- | --- | --- | --- | --- | --- |
| **Site^c^** |  |  |  |  |  |  |
| London  vs. Cambridge | 0.24  (-0.02 to 0.5) | 0.46**  (0.11 to 0.8) | 0.36*  (0.08 to 0.63) | -0.2  (-0.51 to 0.1) | 0.09  (-0.22 to 0.4) | 0.14  (-0.13 to 0.43) |
| Amsterdam  v. Gouda and Voorhout | 0.17  (-0.17 to 0.21) | -0.12  (-0.43 to 0.18) | 0.26  (-0.05 to 0.57) | 0.46**  (0.202 to 0.717) | 0.04  (-0.26 to 0.33) | -0.1  (-0.37 to 0.17) |
| Palermo  v. Verona, Bologna | 0.06  (-0.11 to 0.24) | 0.04  (-0.14 to 0.23) | 0.06  (-0.15 to 0.28) | 0.09  (-0.09 to 0.27) | -0.03  (-0.2 to 0.13) | -0.17*  (-0.32 to -0.02) |
| 20th arrondissement of Paris,  Paris (Val-de-Marne)  v. Puy-de-Dôme | -0.24  (-0.79 to 0.32) | 0.08  (-0.74 to 0.57) | 0.03  (-0.66 to 0.59) | 0.6  (-0.11 to 1.32) | 0.44  (-0.14 to 1) | -0.25  (-0.81 to 0.29) |
| Barcelona, Valencia,  Madrid(Vallecas),  v. Oviedo, Santiago, Cuenca | 0.62***  (0.37 to 0.88) | -0.48**  (-0.77 to -0.19) | -0.38*  (-0.69 to -0.07) | -0.07  (-0.32 to 0.17) | 0.05  (-0.28 to 0.18) | -0.03  (-0.25 to 0.19) |

a. Brazil excluded from this analysis as only a single setting was part of the EU-GEI study

b. The analyses were controlled for age, gender, ethnicity, diagnosis, and type of assessment (interview vs. case records)

c. Population density was dichotomized at its median for defining urban and less urban settings.

**Supplementary Figure S1**

Path diagrams of the five theory-based models of psychopathology**^a^**

**
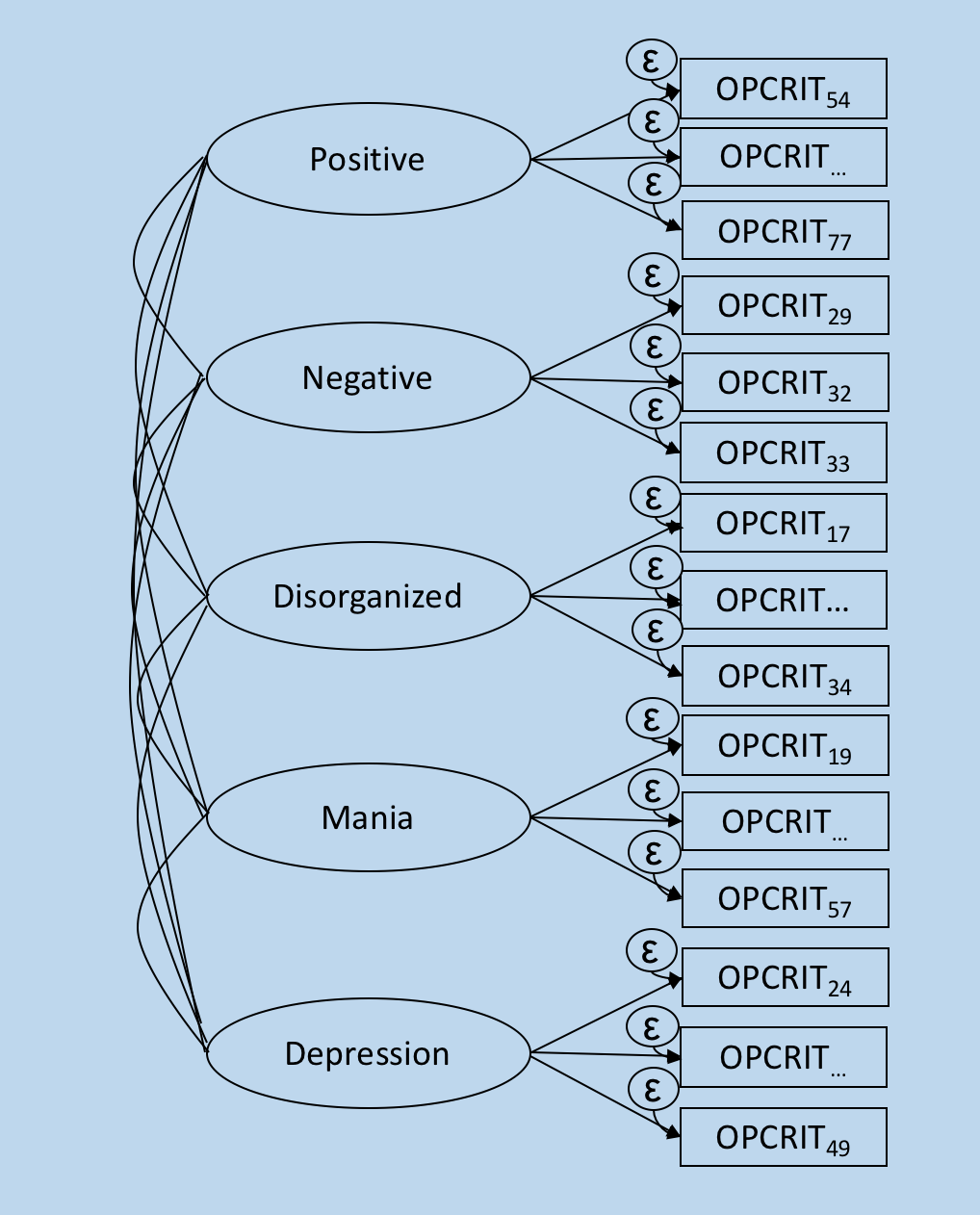
**

*Model A Model B Model C*


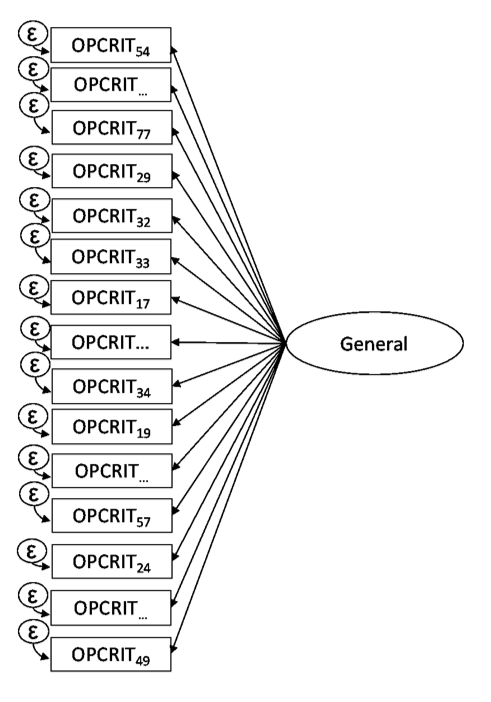
 **
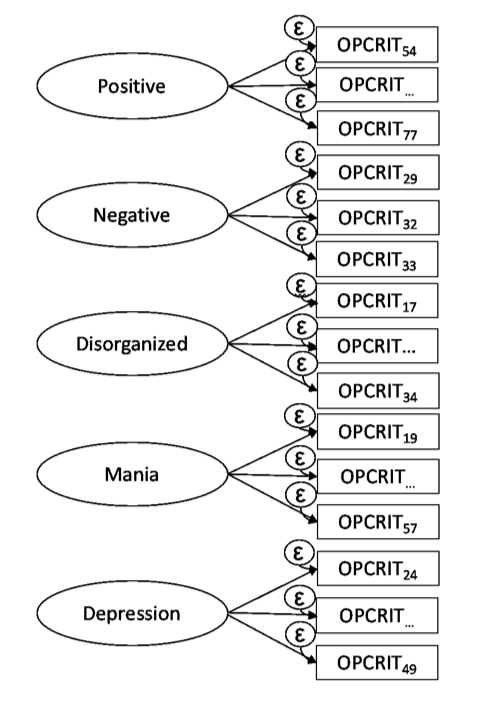
**
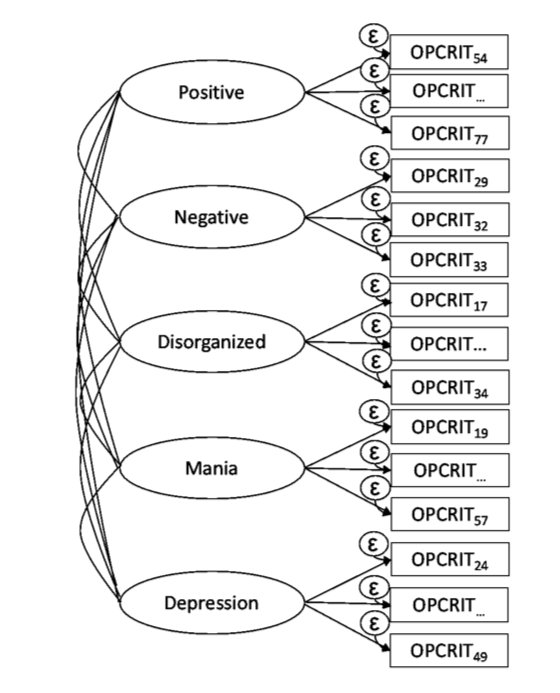


*Model D Model E
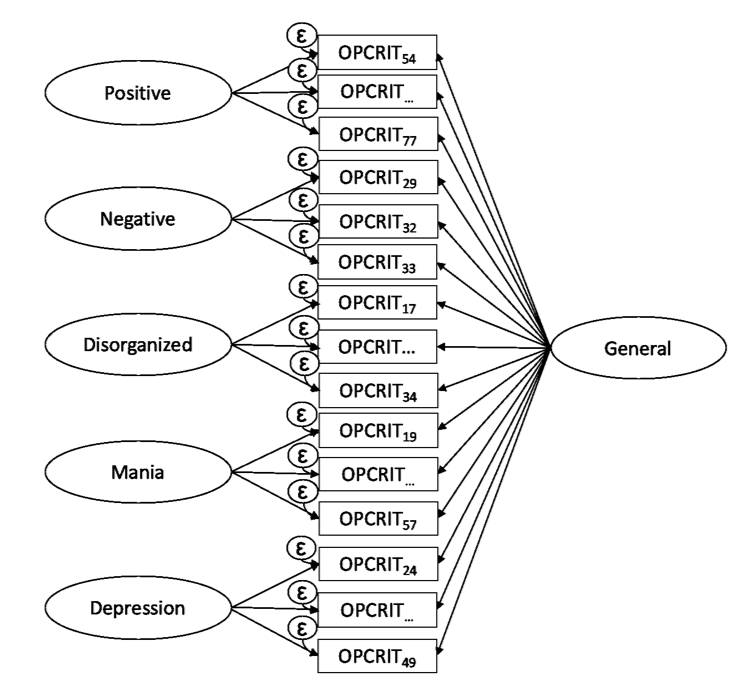

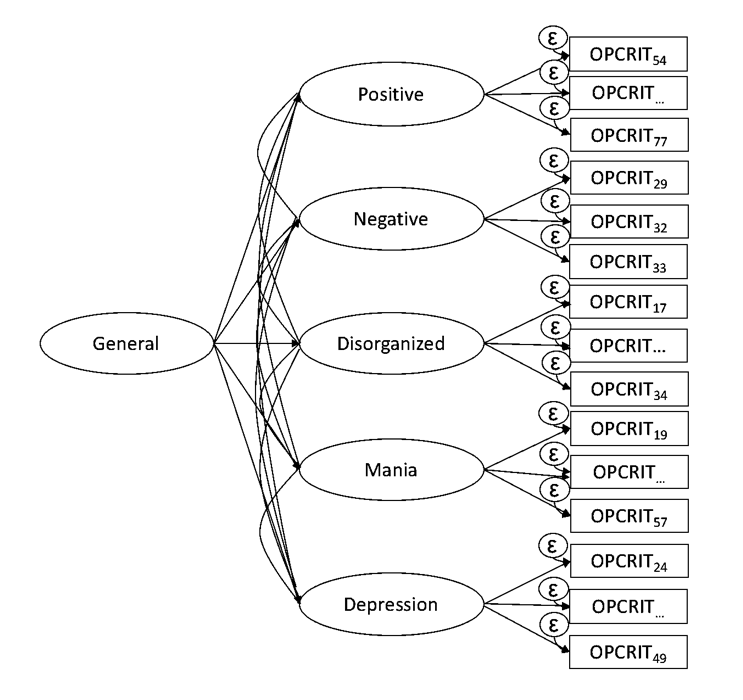
*

(▭) Observed symptoms (OPCRIT items); (Ö) Unobserved variables (latent factors); (⭢) item loading on latent factors; (ε) item error variance; G, general psychosis factor; Specific symptom factors: DEP, Depression; MAN, Mania; DIS, Disorganization; NEG, Negative; POS, Positive. OPCRIT item numbers are showed in Tables S1; for simplicity, only three items for each latent factor are presented in the diagrams.

a. Explanatory note: *Model A*: unidimensional model with one unique general factor; *Model B*: multidimensional model with five uncorrelated specific factors; *Model C*: multidimensional model with five correlated specific factors; *Model D*: bifactor model with one general factor and five uncorrelated specific factors; Model *E*: hierarchical model with five correlated first-order specific factors and one general second-order factor

**Supplementary Figure S2**

Symptom profiles for general and specific symptom dimensions by ICD-based diagnostic category^a^

**
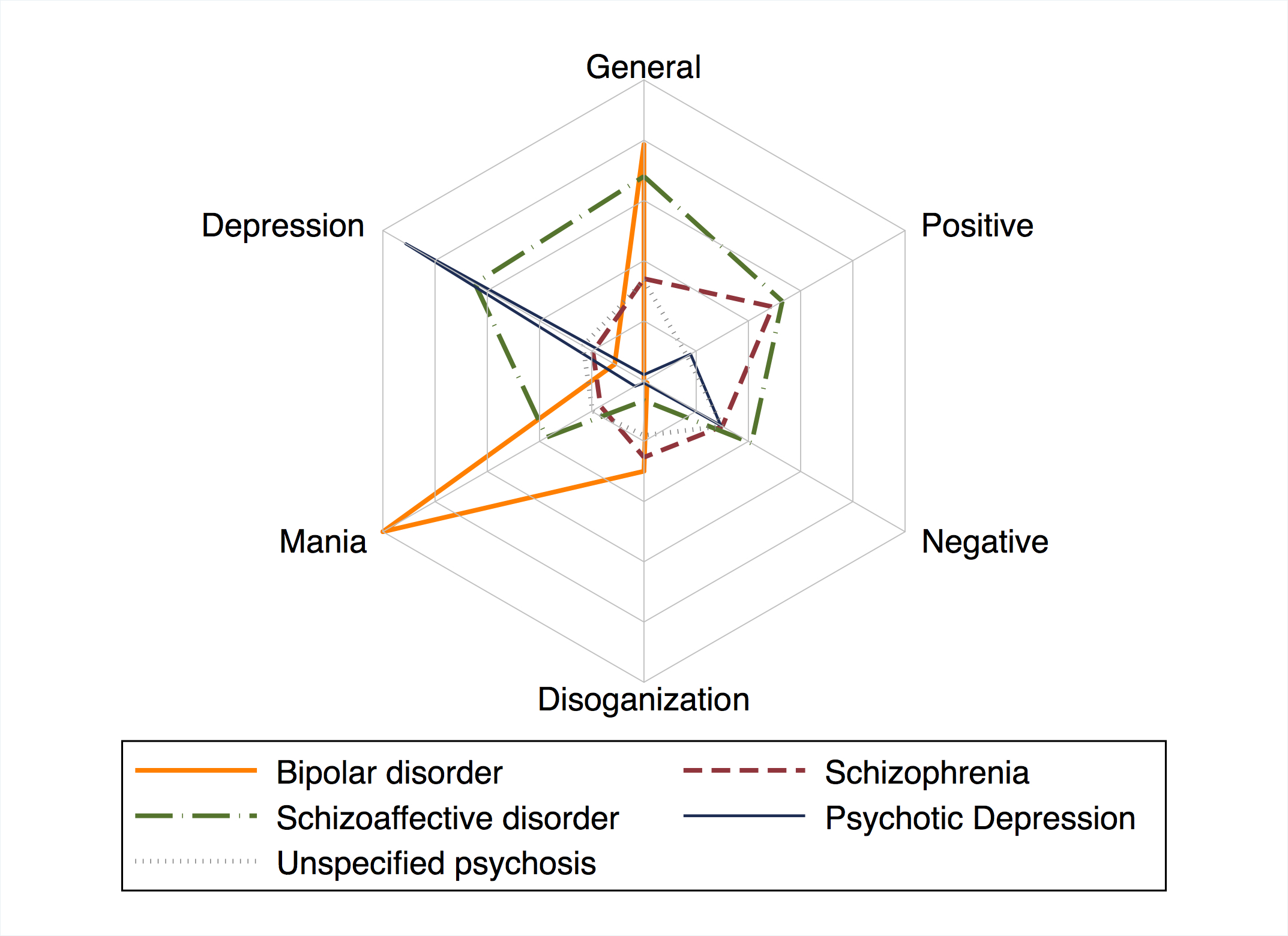
**

a. Explanatory note: Predicted symptom dimension scores by ICD-based diagnostic categories. The continuous symptom dimension scores were computed using the function ‘FSCORES’ in M*plus* (setting mean=0 and standard deviation=1), and used as the outcome variable in the model.

**Supplementary Figures S3.1 and S3.2**

Diagnostic classification accuracy of general and specific symptom dimensions compared with a classification by chance

**Supplementary figure S3.1^a,c^**

**
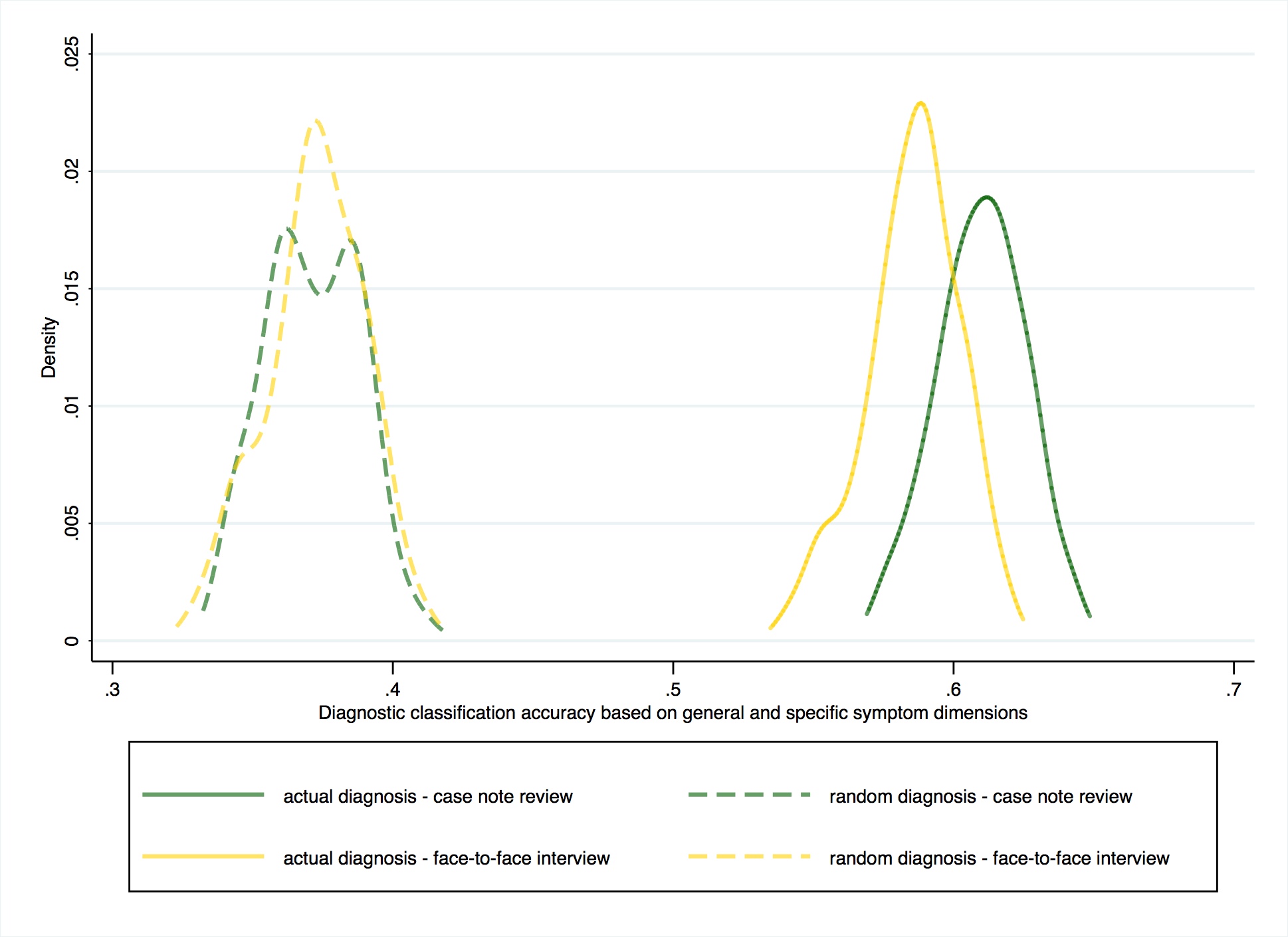
**

**Supplementary figure S3.2^b,c^**


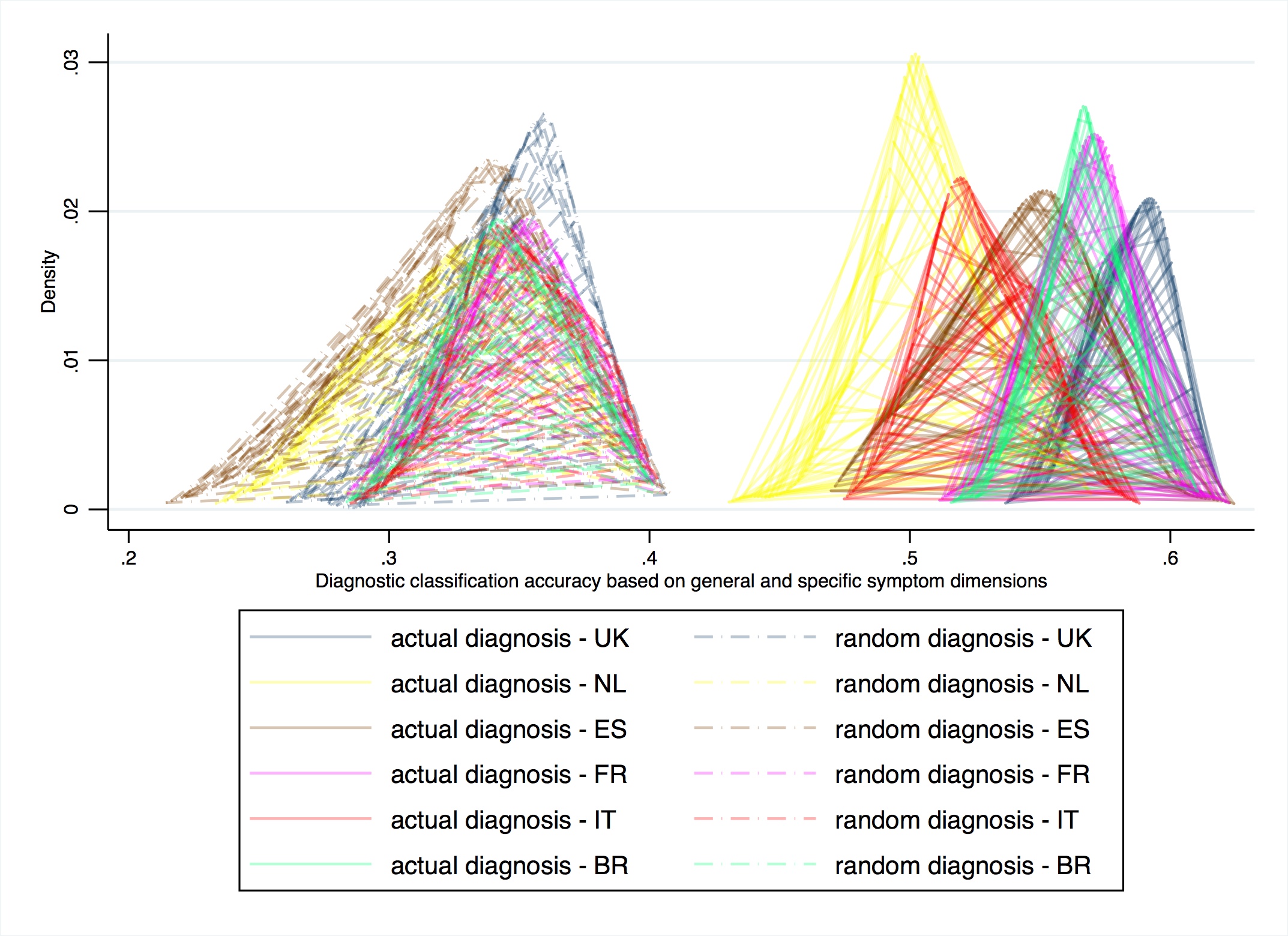


1. Explanatory note 1: Figure S3.1 shows the density distribution (*y-axis*) of the subjects classified in the correct RDC diagnosis (*x-axis*) on the basis of general and specific symptom dimensions scores. Classification accuracy for subjects with psychopathology rating based on face-to-face interview (95% CI 0.54-0.63), and based on the case note review (95% CI 0.56-0.65), is compared with a classification by chance (95% 0.32-0.41).
2. Explanatory note 2: Figure S3.2 shows the density distribution (*y-axis*) of the subjects allocated in the correct RDC diagnosis (*x-axis*) in United Kingdom (UK), the Netherland (NL), Spain (ES), France (FR), Italy (IT), and Brazil (BR). Density peaks to the right of the plot indicates a better accuracy. Classification of subjects based on general and specific symptom dimensions scores was more accurate than a classification by chance in all the countries.
3. Explanatory note 3: Multinomial ROC analysis was composed of two steps. Firstly, we ran B=100 bootstrapped multinomial regression models, predicting RDC-based diagnoses on each of the six dimension scores in a random set of patients. For each model, the quota of the subjects who were correctly classified was determined and annotated. In a second step, we ran B=100 bootstrapped multinomial regression models in each random set of patients, but this time after shuffling RDC diagnoses prior to modelling (under the null hypothesis that symptom dimension scores had no prediction power). Based on kernel density estimation, we therefore obtained 1) the density distribution of the patients correctly allocated into the diagnostic categories based on general and specific symptom dimensions scores (*actual diagnosis*); and 2) the density distribution of the patients allocated into the diagnostic categories by chance (*random diagnosis*). Based on the difference of the two distributions, we may inform on the ability of general and specific symptom dimensions to correctly classify individuals into diagnostic categories.
